# A Plasma-Derived Protein-Metabolite Multiplexed Panel for Early-Stage Pancreatic Cancer

**DOI:** 10.1093/jnci/djy126

**Published:** 2018-08-18

**Authors:** Johannes F Fahrmann, Leonidas E Bantis, Michela Capello, Ghislaine Scelo, Jennifer B Dennison, Nikul Patel, Eunice Murage, Jody Vykoukal, Deepali L Kundnani, Lenka Foretova, Eleonora Fabianova, Ivana Holcatova, Vladimir Janout, Ziding Feng, Michele Yip-Schneider, Jianjun Zhang, Randall Brand, Ayumu Taguchi, Anirban Maitra, Paul Brennan, C Max Schmidt, Samir Hanash

**Affiliations:** 1 Department of Clinical Cancer Prevention, The University of Texas MD Anderson Cancer Center, Houston, TX; 2 Department of Biostatistics, The University of Texas MD Anderson Cancer Center, Houston, TX; 3 Department of Translational Molecular Pathology, The University of Texas MD Anderson Cancer Center, Houston, TX; 4 Department of Pathology, The University of Texas MD Anderson Cancer Center, Houston, TX; 5 International Agency for Research on Cancer (IARC), Lyon, France; 6 Department of Cancer Epidemiology and Genetics, Masaryk Memorial Cancer Institute, Brno, Czech Republic; 7 Regional Authority of Public Health in Banska Bystrica, Banska Bystrica, Slovakia; 8 Catholic University, Faculty of Healthy, Ružomberok, Slovakia; 9 Institute of Public Health and Preventive Medicine, 2nd Faculty of Medicine, Charles University, Prague, Czech Republic; 10 Faculty of Medicine, Palacky University, Olomouc, Czech Republic; 11 Department Surgery, Indiana University School of Medicine, Indianapolis, IN; 12 Department of Epidemiology, Fairbanks School of Public Health, Indiana University, Indianapolis, IN; 13 Department of Medicine, University of Pittsburgh, Pittsburgh, PA

## Abstract

**Background:**

We applied a training and testing approach to develop and validate a plasma metabolite panel for the detection of early-stage pancreatic ductal adenocarcinoma (PDAC) alone and in combination with a previously validated protein panel for early-stage PDAC.

**Methods:**

A comprehensive metabolomics platform was initially applied to plasmas collected from 20 PDAC cases and 80 controls. Candidate markers were filtered based on a second independent cohort that included nine invasive intraductal papillary mucinous neoplasm cases and 51 benign pancreatic cysts. Blinded validation of the resulting metabolite panel was performed in an independent test cohort consisting of 39 resectable PDAC cases and 82 matched healthy controls. The additive value of combining the metabolite panel with a previously validated protein panel was evaluated.

**Results:**

Five metabolites (acetylspermidine, diacetylspermine, an indole-derivative, and two lysophosphatidylcholines) were selected as a panel based on filtering criteria. A combination rule was developed for distinguishing between PDAC and healthy controls using the Training Set. In the blinded validation study with early-stage PDAC samples and controls, the five metabolites yielded areas under the curve (AUCs) ranging from 0.726 to 0.842, and the combined metabolite model yielded an AUC of 0.892 (95% confidence interval [CI] = 0.828 to 0.956). Performance was further statistically significantly improved by combining the metabolite panel with a previously validated protein marker panel consisting of CA 19–9, LRG1, and TIMP1 (AUC = 0.924, 95% CI = 0.864 to 0.983, comparison DeLong test one-sided *P=* .02).

**Conclusions:**

A metabolite panel in combination with CA19-9, TIMP1, and LRG1 exhibited substantially improved performance in the detection of early-stage PDAC compared with a protein panel alone.

Pancreatic ductal adenocarcinoma (PDAC) is the third leading cause of cancer-related mortality, with an overall five-year survival rate of only approximately 8% (1). Diagnosis of PDAC at an early stage is uncommon, with the majority of patients presenting with locally advanced or metastatic disease (2). Early detection of PDAC will lead to improved survival. CA19-9 has limited performance as a PDAC biomarker, particularly in the prediagnostic setting (3–5). Moreover, it is noninformative in approximately 10% of subjects with fucosyltransferase deficiency (6). Consequently, there is a critical need for additional markers that display collectively higher sensitivity and specificity for reliable detection of early-stage PDAC.

Previously, we identified and sequentially validated two additional proteins, TIMP1 and LRG1, that complemented CA19-9 in distinguishing early-stage PDAC from healthy subjects (7). Although classification performance improved relative to CA19-9 alone, further improvements to increase sensitivity at high specificity are desirable given the low incidence of PDAC.

More than 95% of all existing diagnostic clinical assays look for small molecules (eg, <1200 Da) (8). CA19-9 is in fact a carbohydrate small molecule antigen. Clinical metabolomics is an emerging field, and untargeted metabolomics represents a global unbiased approach for the profiling of small molecules that is increasingly being implemented in biomarker discovery from a variety of human biofluids and tissues (9–12).

In the current study, we applied an untargeted metabolomics approach to develop a plasma-derived metabolite biomarker panel for PDAC. Our fixed biomarker panel was subsequently blindly validated in an independent test cohort consisting of 39 resectable PDAC cases and 82 matched healthy controls. We additionally evaluated whether a protein-metabolite multiplexed panel consisting of our metabolite panel plus a previously validated protein marker panel consisting of CA19-9, TIMP1, and LRG1 would improve classification performance compared with CA19-9 or the protein panel alone.

## Methods

### Study Population

**Figure 1.**
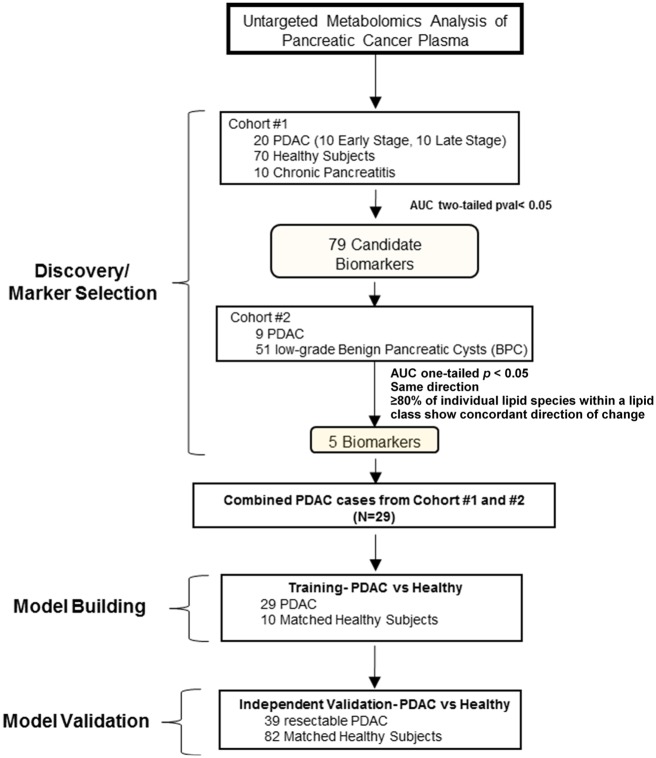
Schematic of study design. AUC = area under the curve; PDAC = pancreatic ductal carcinoma.

Detailed information is provided in the Supplementary Methods (available online). All human blood samples were obtained following institutional review board approval, and patients provided written informed consent. For discovery studies, plasma samples from 20 patients with PDAC, 70 healthy controls, and 10 patients with chronic pancreatitis were obtained from the Evanston Hospital and MD Anderson Cancer Center (Cohort 1). Plasma samples obtained from the Indiana University School of Medicine, consisting of 51 patients with low–dysplastic grade pancreatic cysts and nine patients with invasive intraductal papillary mucinous neoplasms (IPMNs) were used for biomarker sequential selection and initial validation (Cohort 2). An independent plasma sample set for blinded testing of the combined biomarker panel(s) was obtained from the International Agency for Research on Cancer and consisted of 39 early-stage PDAC patients and 82 healthy controls (Test Set). Study flow diagram and clinical characteristics of the patients in the discovery cohorts (Cohort 1 and 2) and blinded validation cohort (Test Set) are presented in Figure 1 and Supplementary Tables 1, 2, and 3 (available online).

### Metabolomic Analysis and Enzyme-Linked Immunosorbent Assay

Detailed information regarding metabolomic analyses and enzyme-linked immunosorbent assays for CA19-9, LRG1, and TIMP1 are provided in the Supplementary Methods (available online). Descriptions of cell lines that were profiled using untargeted metabolomics technology are provided in Supplementary Table 4 (available online). Metabolomic profiling was conducted on a Waters Acquity UPLC system with 2D column regeneration configuration (I-class and H-class) coupled to a Xevo G2-XS quadrupole time-of-flight (qTOF) mass spectrometer. Chromatographic separation was performed using HILIC (Acquity UPLC BEH amide, 100 Å, 1.7 µm 2.1 × 100 mm) and C18 (Acquity UPLC HSS T3, 100 Å, 1.8 µm, 2.1 × 100 mm) columns at 45°C. Mass spectrometry data were acquired in sensitivity and positive/negative electrospray ionization mode. The acquisition was carried out with instrument auto gain control to optimize instrument sensitivity over the samples’ acquisition time. Data processing was performed as previously described (13). To adjust for intersite variability, each metabolite was standardized by median-centering the metabolite value for each sample to the median value of the healthy controls. Plasma protein concentrations for CA19-9, LRG1, and TIMP1 were determined as previously described (Supplementary Methods, available online) (7).

### Gene Expression Data and Networks

Gene expression for the Badea data set (14) was downloaded from the Oncomine database (15). The Badea data set was chosen as it contains adequate sample size with respect to controls (n = 39) as compared with other data sets, such as The Cancer Genome Atlas, and because it allows for comparison of altered mRNA expression between PDAC and matched adjacent control tissues, thereby mitigating intersubject variations. Networks were visualized using cytoscape (16).

### Statistical Analyses

Receiver operating characteristic (ROC) curve analysis was performed to assess the performance of biomarkers in distinguishing PDAC cases from healthy controls and subjects diagnosed with benign pancreatic disease (chronic pancreatitis or pancreatic cysts). Detailed methods regarding the development of the biomarker panel are included in the Supplementary Methods (available online). Briefly, model building was based on a logistic regression model using the logit link function. The estimated AUC of the proposed metabolite panel was derived by using the empirical ROC estimator of the linear combination corresponding to the aforementioned model. The protein-metabolite multiplexed panel of metabolites and proteins was developed by combining the two underlying panels (metabolite and protein) as two composite markers with the use of a logistic regression model that incorporates the logit link function. For the testing set, all *P* values reported for individual marker AUC testing are Wilcoxon based, whereas for comparing two correlated AUCs, the Delong test was employed. We note that for the Test Set, the coefficients of the trained logistic regression model are considered fixed and known; hence traditional tests can be applied. For comparison of mRNA expression from the Badea data set, *P* values were calculated using paired two-sided *t* tests. A *P* value of less than .05 was considered statistically significant under all circumstances.

## Results

### Identification of Pancreatic Cancer Metabolite Biomarkers and Model Development

**Figure 2.**
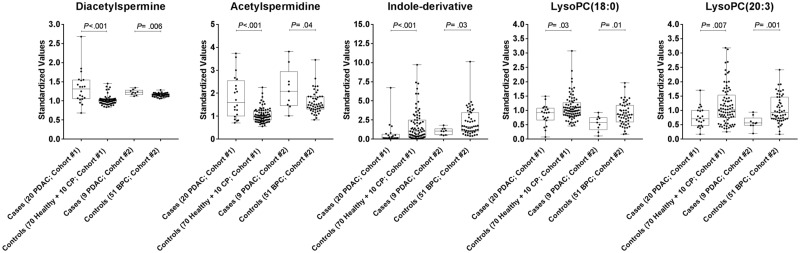
Distributions of the five metabolite biomarkers in discovery cohorts 1 and 2. Box and whisker plots are shown for individual metabolites. Values represent the ratios of the respective metabolite relative to historical quality control reference measurement (see the “Methods”). Statistical significance was determined by two-sided Wilcoxon rank-sum test. PDAC = pancreatic ductal carcinoma.

Untargeted metabolomics analysis was conducted on a discovery cohort (Cohort 1) consisting of 20 PDAC cases (10 early and 10 late stage) and 80 controls (70 healthy subjects and 10 subjects with chronic pancreatitis) (Figure 1). Candidate biomarkers were initially selected based on statistically significant ROC AUCs (two-sided Wilcoxon rank-sum test *P* < .05); candidate markers were subsequently filtered against a second independent cohort (Cohort 2) consisting of nine PDAC (five early and four late stage) and 51 subjects with benign pancreatic cysts (BPCs) (Figure 1) based on statistically significant ROC AUCs (two-tailed Wilcoxon rank-sum test *P* < .05) and maintaining the same direction of change (Supplementary Tables 5 and 6, available online). In the case of individual lipid species, to mitigate nonspecificity due to external factors such as dietary patterns or randomness, emphasis was given to those lipids that showed uniformity in the performance characteristics among the entire lipid class (ie, >80.0% of the detected individual lipids in a given lipid class exhibited concordant increases/decreases in cases relative to controls) (Supplementary Figure 1, available online). Five metabolites were selected that met the aforementioned criteria that consisted of (N1/N8)-acetylspermidine (AcSperm), diacetylspermine (DAS), lysophosphatidylcholine(18:0) (LPC(18:0)), LPC(20:3), and an indole-derivative (Figure 2; fragmentation spectrum is provided in Supplementary Figure 2, available online).

**Figure 3.**
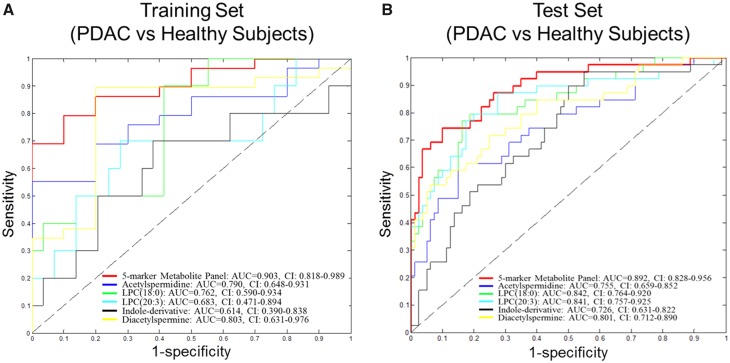
Areas under the curve (AUCs) of individual metabolites and metabolite panels in the Training and Test Sets. **A)** Receiver operating characteristic (ROC) curves for individual metabolites and the five-marker metabolite panel for distinguishing pancreatic ductal adenocarcinoma (PDAC; n = 29) from healthy subjects (n = 10). **B)** ROC curves for individual metabolites and the five-marker metabolite panel for distinguishing resectable PDAC (n = 39) from healthy subjects (n = 82; Test Set 1). AUC = area under the curve; CI = confidence interval.

Next, we developed a biomarker panel combination rule for PDAC based on a logistic regression model. The model was developed using data from the Training Set (Figure 1). In the comparison of PDAC vs healthy subjects, the resulting panel of AcSperm + DAS + LPC(18: 0) + LPC(20: 3) + indole-derivative yielded an AUC of 0.903 (95% confidence interval [CI] = 0.818 to 0.989), which exhibited 69.0% sensitivity at 99.0% specificity (Figure 3A).

### Testing of Metabolite Biomarker Panel in an Independent Test Set of Resectable PDAC Plasma Samples

Blinded validation of the five metabolites individually and as a panel was performed in an independent set of plasma samples consisting of 39 resectable PDAC cases and 82 matched healthy controls (Test Set). All five biomarkers yielded statistically significant differences (one-tailed *P* < .001) in PDAC cases vs healthy controls, with individual AUCs ranging from 0.726 to 0.842 (Table 1). The fixed logistic regression model for the five-metabolite panel yielded an AUC of 0.892 (95% CI = 0.828 to 0.956), with 66.7% sensitivity at 95.0% specificity (Figure 3B, Table 1).

**Table 1.** Performance of individual metabolite markers and metabolite panel in the validation cohort (Test Set)

Metabolite	AUC (95% CI)	P*	Specificity†, %	Sensitivity‡, %
Indole-derivative§	0.726 (0.631 to 0.822)	<.001	11.3	23.1
LPC(18:0)§	0.842 (0.764 to 0.920)	<.001	26.3	51.3
LPC(20:3)§	0.841 (0.757 to 0.925)	<.001	11.3	48.7
Acetylspermidine	0.755 (0.659 to 0.852)	<.001	27.5	33.3
Diacetylspermine	0.801 (0.712 to 0.890)	<.001	27.5	51.3
5-marker metabolite panel	0.892 (0.828 to 0.996)	<.001	43.3	66.7

* *P* values for corresponding area under the curve results, Wilcoxon rank-sum test one-sided. AUC = area under the curve; CI = confidence interval; LPC = lysophosphatidylcholine.

† % specificity at 95% sensitivity.

‡ % sensitivity at 95% specificity.

§ AUCs <0.5 are flipped (ie, equivalent to 1-AUC due to reverse ordering).

### Comparison of Classifier Performance of Metabolite Plus Protein Markers CA19-9, TIMP1, and LRG1 vs Classifier Performance CA19-9 or Protein Markers Alone

**Figure 4.**
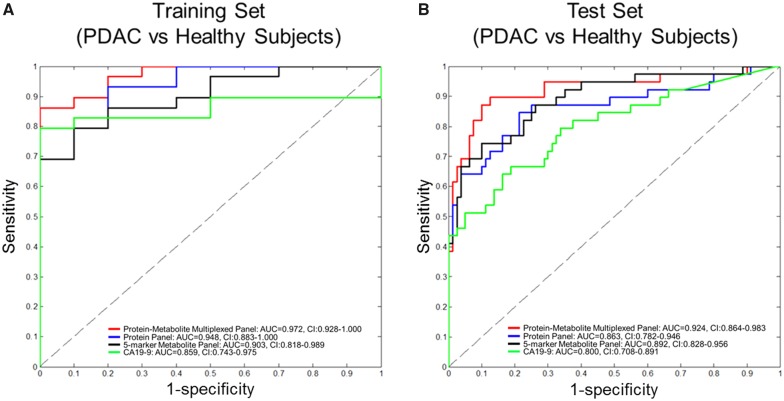
Areas under the curve (AUCs) for protein-metabolite multiplexed panel, five-marker metabolite-panel, three-marker protein panel, and CA19-9. **A and B)** Receiver operating characteristic (ROC) curves for protein-metabolite multiplexed panel, five-marker metabolite-panel, three-marker (LRG, TIMP1, CA19-9) protein panel, and CA19-9 in the Training Set (29 PDAC vs 10 healthy subjects) and independent Test Set (39 PDAC vs 82 healthy subjects). AUC = area under the curve; CI = confidence interval.

Previously, we identified and sequentially validated two additional proteins, TIMP1 and LRG1, capable of complementing CA19-9 in distinguishing PDAC from healthy subjects (7). We therefore interrogated whether a protein-metabolite multiplexed panel consisting of our protein markers (CA19-9, TIMP1, and LRG1) together with our metabolite panel would improve classification performance as compared with CA19-9 or our protein panel alone in distinguishing PDAC from healthy subjects. When applied to the Training Set (29 PDAC vs 10 matched EHO healthy controls), the protein-metabolite multiplexed panel yielded an AUC of 0.972 (95% CI = 0.928 to 1.000). This estimate was statistically significantly better than CA19-9 alone (AUC = 0.859, 95% CI = 0.743 to 0.975, comparison DeLong test one-tailed *P* = .03) but not the protein panel (AUC = 0.948, 95% CI = 0.883 to 1.000, comparison DeLong test one-tailed *P *=* *.11) (Figure 4A). During blinded validation, the resulting protein-metabolite multiplexed panel yielded an AUC of 0.924 (95% CI = 0.864 to 0.983) (Figure 4B). This estimate was statistically significantly greater than either CA19-9 alone (AUC = 0.800, 95% CI = 0.708 to 0.891, comparison DeLong test one-tailed *P *<* *.001) or the protein panel alone (AUC = 0.863, 95% CI = 0.782 to 0.946, comparison DeLong test one-tailed *P* = .02) (Figure 4B). Youden-based cutoffs and associated pairs of sensitivity and specificity attained at these cutoffs for the protein-, metabolite-, and protein-metabolite multiplexed panel for the Test Set are provided in Supplementary Table 7 (available online).

### Polyamine Metabolism and PDAC

**Figure 5.**
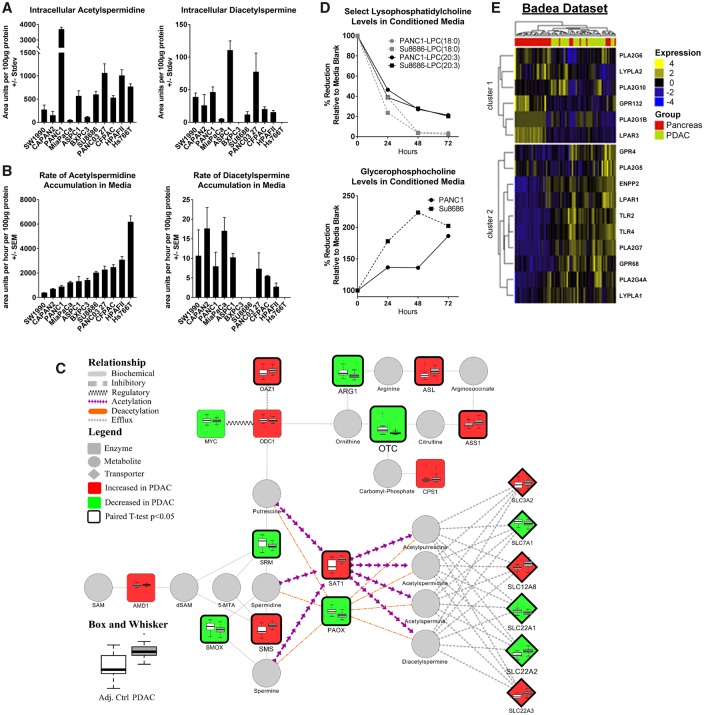
Polyamine and lipid metabolism in PDAC. **A)** Abundances (area units +/- SD) of N1/N8-acetylspermidine or diacetylspermine in cell lysates of 11 PDAC cell lines. **B)** Abundance (area units +/- SD) of N1/N8-acetylspermidine or diacetylspermine in serum-free media collected one, two, four, and six hours post conditioning from 11 PDAC cell lines. **C)** Network displaying enzymes involved in the biosynthesis of polyamines and their acetylated derivatives. Node color (light gray = decreased; dark gray = increased) and size depict the direction and magnitude of change in mRNA expression of respective enzymes between PDAC and adjacent control tissue. Thickened node border illustrates statistical significance (two-sided paired *t* test *P* < .05). Box and whisker plots illustrate the distribution of mRNA expression for the respective enzyme between PDAC and adjacent control tissue. mRNA expression data were obtained from Oncomine (15) and are based on the Badea data set (14). **D)** Percent change in serum-containing media composition of lysophosphatidylcholine(18:0), lysophosphatidylcholine(20:3), and glycerophosphocholine in PANC1 and SU8686 PDAC cell lines following 24, 48, and 72 hours of culturing. **E)** Heat map depicting mRNA expression of enzymes and surface receptors known to directly participate in the metabolism or binding of lysophosphatidylcholines between PDAC and adjacent control tissue. Data were obtained from Oncomine (15) and are based on the Badea data set (14). Unsupervised clustering was performed using Euclidean distance with Ward’s method.

We explored further whether PDAC cells secreted AcSperm and DAS. We analyzed cell lysates and serum-free conditioned media from 11 PDAC cell lines. Metabolomic analysis of cell lysates revealed detectable levels of AcSperm in all 11 PDAC cell lines, whereas DAS was detected in nine out of 11 PDAC cell lines (Figure 5A). Analysis of conditioned media indicated positive rates (area units per hour per 100 µg protein) of AcSperm accumulation in all 11 cell lines. Positive rates of DAS accumulation were observed in eight of the 11 cell lines (Figure 5B). Exploration of mRNA expression of polyamine-related enzymes in the Badea data set (14) indicated statistically significant (two-sided paired *t* test *P*<.001) PDAC-associated elevations in spermine synthase (*SMS*) and spermidine/spermine acetyltransferase (*SAT1*) compared with adjacent control tissue, whereas spermidine synthase (*SRM*), polyamine oxidase (*PAOX*), and spermine oxidase (*SMOX*) were statistically significantly reduced (two-sided paired *t* test *P* < .001, <.001, and .002, respectively), collectively suggesting increased acetylation of polyamines and subsequent secretion rather than their oxidation (Figure 5C).

### Lipids Metabolism and PDAC

To determine whether PDAC cells catabolize/scavenge extracellular lipids, we examined the lipid composition of serum-containing media from PANC1 and Su8686 cells 24, 48, and 72 hours postconditioning. Our analysis indicated time-dependent reductions in several lysophospholipids (Supplementary Figure 3, available online), including LPC(18:0) and LPC(20:3) (Figure 5D). Concomitantly, glycerophosphocholine, a degradation product of LPCs, exhibited a time-dependent increase in conditioned media (Figure 5D), collectively implicating active catabolism of extracellular LPCs. Evaluation of mRNA expression for enzymes involved in the generation and catabolism of LPCs indicates numerous PDAC-associated elevations including autotaxin (*ENPP2*) and lysophospholipase *LYPLA1* that were statistically significant (two-sided paired *t* test *P* < .001) relative to adjacent control tissue in the Badea data set (Figure 5E). Moreover, relative to adjacent control tissue, PDAC tissue exhibited statistically significantly higher mRNA expression of genes encoding for cell surface receptors that are known to be activated by LPCs, including *GPR4, GPR68, TLR2, TLR4*, and *LPAR1* (two-sided paired *t* test *P* = .05, <.001, <.001, <.001, and .03, respectively) (Figure 5E).

## Discussion

Using an untargeted metabolomics approach, we have identified and validated a five-marker metabolite-derived biomarker panel for distinguishing PDAC from healthy subjects. Importantly, a protein-metabolite multiplexed panel consisting of metabolite and previously identified protein biomarkers (7) resulted in improved classification performance relative to either the metabolite, CA19-9, or protein panel alone. Thus the combination of different biomarker types yielded superior results relative to a single biomarker type.

Of note, we did not observe differences in plasma branched-chain amino acids (BCAA) between cases and controls, as was previously reported in prediagnostic samples (17). In the prior reported study, the predictive value of BCAAs was most prominent two to five years before diagnosis, with levels returning toward baseline closer to diagnosis (17), consistent with our observation of no differences in plasma BCAAs in samples taken at the time of diagnosis.

Altered polyamine metabolism has been linked to tumorigenesis and hyperproliferative disorders, being intimately involved in cell cycle progression (18). Polyamine synthesis is regulated by the rate-limiting enzymes ODC1 and AMD1, whereas their catabolism is regulated by SAT1 (18,19). Previous findings indicated increased abundance of putrescine and AcSperm in pancreatic carcinomas as compared with histologically unaffected pancreas (20). Conversely, many polyamines including AcSperm were elevated in sera of cases compared with healthy controls (20). Our findings and those of others indicate amplification of polyamine catabolism, a notion that is reflected in plasmas of subjects with PDAC. Notably, our in vitro findings demonstrated an inverse association between rates of AcSperm and DAS accumulation in conditioned media, collectively highlighting an intrinsic heterogeneity that exists among PDAC with respect to polyamine catabolism. These findings also provide merit for analyzing both polyamine markers as biomarkers for PDAC. Conversely, the elevation of DAS is not uniquely attributed to pancreatic cancer (9,21) inherently, suggesting a more general role in its broader utility as a screening marker for cancer.

Other previous studies indicated that plasma LPCs are statistically significantly lower in PDAC relative to healthy controls (10,22) or subjects with chronic pancreatitis (23), consistent with our findings. Our cell line data indicated that PDAC cells catabolize lysophospholipids, a notion that is supported by gene expression data in the Badea data set. However, PDAC alone cannot account for the reduction in plasma LPC levels entirely, particularly in the early stages of the disease. It is plausible that reductions in plasma LPCs may be a reflection of both increased catabolism by cancer cells and altered liver function that co-occurs with disease (24–26).

A limitation for studies aimed at discovery and/or validation of pancreatic cancer early detection is accessibility to adequate number of patients with early-stage (stage IA) disease or with premalignant lesions. To this end, we acknowledge that the current panel requires further validation in independent cohorts. We additionally recognize that the current multiplexed protein-metabolite panel has not been tested for its ability to distinguish PDAC from other benign conditions or malignancies, an important consideration when fully assessing its utility as a screening tool.

In conclusion, we have developed and validated a metabolite-derived biomarker panel for early-stage PDAC that complements our previously identified protein-based biomarker panel. Given the low prevalence of PDAC, our current multimarker signature would be best suited for screening programs targeting high-risk subjects rather than the average-risk population. These include individuals older than age 50 years with new-onset diabetes mellitus, asymptomatic kindred of high-risk families, subjects with chronic pancreatitis, and patients incidentally diagnosed with mucin-secreting cysts of the pancreas (27–30). Further improvement in performance for early detection application may result from expansion of the panel to include other marker types, such as circulating tumor DNA and autoantibodies to tumor antigens (31–33).

## Funding

This work was supported by the MD Anderson’s Moonshot Program, the National Cancer Institute Early Detection Network, the Pancreatic Cancer Action Network, two faculty fellowships from The University of Texas MD Anderson Cancer Center Duncan Family Institute for Cancer Prevention and Risk Assessment (JFF and MC), Stand Up to Cancer- Lustgarten Foundation (SU2C-AACR-DT25-17), and the National Institutes of Health (NIH) R21 (IR32CA209366-01). Additional support was acquired from NIH-R21 (1R21CA209366-01; CMS, MY, and JJ), NIH-U01 EDRN (1U01CA200468; CMS, MY, and AM), and NIH-U01 (1U01CA196403; CMS, MY, and AM). The case–control study that provided the Test Set was funded by the National Cancer Institute at the National Institutes of Health (R03 CA123546-02), the Development of Research Organization of the Ministry of Health of the Czech Republic (MMCI, 00209805; IGA MZ N, 9422-3 and 8090-3), and the Ministry of Health of the Slovak Republic for the Epidemiological Study on Pancreatic Cancer, ESNAP (Epidemiological Study on Pancreatic Cancer, Regional Authority of Public Health in Banska Bystrica, MZSR2007/17-RUVZBB-02).

## Notes

Affiliations of authors: Department of Clinical Cancer Prevention (JFF, MC, JBD, NP, EM, JV, DLK, SH), Department of Biostatistics (LEB, ZF), Department of Translational Molecular Pathology (AT), and Department of Pathology (AM), The University of Texas MD Anderson Cancer Center, Houston, TX; International Agency for Research on Cancer (IARC), Lyon, France (GS, PB); Department of Cancer Epidemiology and Genetics, Masaryk Memorial Cancer Institute, Brno, Czech Republic (LF); Regional Authority of Public Health in Banska Bystrica, Banska Bystrica, Slovakia (EF); Catholic University, Faculty of Healthy, Ružomberok, Slovakia (EF); Institute of Public Health and Preventive Medicine, 2nd Faculty of Medicine, Charles University, Prague, Czech Republic (IH); Faculty of Medicine, Palacky University, Olomouc, Czech Republic (VJ); Department Surgery, Indiana University School of Medicine, Indianapolis, IN (MYS, CMS); Department of Epidemiology, Fairbanks School of Public Health, Indiana University, Indianapolis, IN (JZ); Department of Medicine, University of Pittsburgh, Pittsburgh, PA (RB).

The funders had no role in the design of the study; the collection, analysis, or interpretation of the data; the writing of the manuscript; or the decision to submit the manuscript for publication.

RB receives research support and has received honoraria from Invitae and is a consultant for Myriad Genetics. AM is a consultant for Celgene and receives research support from Opsona Therapeutics. The other authors have no conflicts of interest to report.

## Supplementary Material

Supplementary Data
