# Supplementary material for: A Plasma-Derived Protein-Metabolite Multiplexed Panel for Early-Stage Pancreatic Cancer
Source: J Natl Cancer Inst. 2018 Aug 18;111(4):372–9. doi: 10.1093/jnci/djy126 (PMC6449169; doi:10.1093/jnci/djy126)

SUPPLEMENTARY MATERIAL

Supplementary Methods

Study Population

All human blood samples were obtained following Institutional Review Board approval and informed consent. For discovery studies, plasma samples from 20 patients with PDAC (10 early-stage and 10 late-stage), and 70 healthy controls and 10 patients with chronic pancreatitis were obtained from the Evanston Hospital and MD Anderson Cancer Center (Cohort #1). All chronic pancreatitis samples were collected in an elective setting in the clinic in the absence of an acute flare-up. Healthy control samples (n=60) were drawn from patients enrolled in a biomarker discovery trial to aid low-dose-CT-based screening for lung cancer at the University of MD Anderson. For the biomarker sequential selection series (Cohort #2), we used plasma samples obtained from the Indiana University School of Medicine, consisting of 51 patients with low dysplastic grade pancreatic cyst and 9 patients with PDAC (5 early-stage and 4 late-stage adenocarcinoma). All patients underwent surgical resection of their cystic lesion, and plasma samples were collected prior to surgery. Dysplastic grade was histopathology confirmed after surgical resection and determined according to WHO criteria. An additional independent plasma sample set for testing the combined biomarker panel was obtained from the International Agency for Research on Cancer, consisting of 39 early-stage PDAC and 82 healthy controls (Test Set). Study flow diagram and clinical characteristics of the patients in the validation sets and test set are presented in **Figure 1** and **Supplementary Tables 1, 2 and 3**.

Metabolomic Analysis

Cell Line Metabolomic Experiments

PDAC cell lines were grown in RPMI-1640 with 10% FBS. The identity of each cell line was confirmed by DNA fingerprinting via short tandem repeats at the time of mRNA and total protein lysate preparation using the PowerPlex 1.2 kit (Promega). Fingerprinting results were compared with reference

fingerprints maintained by the primary source of the cell line. Cells were seeded in 6cm dishes (Thermo Scientific) to reach a 70% (50-80%) confluency, 24 hours post initial seeding. Post 24 hours, cell lysates were washed 2x with pre-chilled 0.9% NaCl followed by addition of 1mL of pre-chilled extraction buffer (3:1 isopropanol:ultrapure water) to quench and remove cell media. Cells were then scraped using a 25cm Cell Scraper (Sarstedt) in extraction solvent and transferred to a 1.5mL eppendorf tube. After vortexing briefly, the extracted cell lysates were centrifuged at 4°C for 10 min at 2,000 x g. Thereafter, 1mL of the supernatant containing the extracted metabolites were transferred to 1.5mL Eppendorf tubes and stored in -20°C until needed for metabolomic analysis.

Exometabolome Experiments

Cells were grown in 1ml of DMEM + 10% FBS in 12-well dishes (Costar) to reach a 70% (50-80%) confluency, 24 hours post initial seeding. On the day of the experiment, the cells were washed 2 times with 500µL serum free DMEM (Fisher Scientific) containing 5mM glucose and 0.5mM glutamine. Then, serum free DMEM (300µL) containing 5mM glucose and 0.5mM glutamine was added to each well and cells incubated. After predetermined incubation time (1, 2, 4 and 6 hours) 250µL of the conditioned media was collected. For baseline (T0), 250µL of media was collected directly after the addition of 300µL. All time points were performed in triplicates or quadruplicates. Blank samples containing media only were included and collected at T0 and T6. The 6 hours samples were used to count cell numbers for data normalization. Once all the media samples were collected, the tubes were centrifuged at 2000 x g for 10 min to remove residual debris and the supernatants transferred to 1.5 mL tubes (Eppendorf) and stored in -80°C until use for metabolomics analysis.

Primary Metabolites and Biogenic Amines

Plasma metabolites were extracted from pre-aliquoted EDTA plasma (10 µL) with 30µL of LCMS grade methanol (ThermoFisher) in a 96-well microplate (Eppendorf). Plates were heat sealed, vortexed for 5min at 750 rpm, and centrifuged at 2000 × g for 10 minutes at room temperature. The supernatant (10 µL) was carefully transferred to a 96-well plate, leaving behind the precipitated protein. The supernatant was further diluted with 10 µL of 100 mM ammonium formate, pH3. For Hydrophilic Interaction Liquid

Chromatography (HILIC) analysis, the samples were diluted with 60 µL LCMS grade acetonitrile (ThermoFisher), whereas samples for C18 analysis were diluted with 60 µL water (GenPure ultrapure water system, Thermofisher). Each sample solution was transferred to 384-well microplate (Eppendorf) for LCMS analysis.

For cell lysates, 100 µL (3:1 isopropanol:ultrapure water) was aliquoted into two 300 µL, 96-well plates (Eppendorf) and evaporated to dryness under vacuum. The samples were then reconstituted as follows: for the HILIC assays, the dried samples were dissolved in 65 µL of ACN (Fisher Scientific): 100 mM Ammonium Formate pH3 (9:1) whereas for the reverse phase C18 assays, the dried samples were dissolved in 65 µL of H2O: 100 mM Ammonium Formate pH3 (9:1). The samples were then spun down to remove any insoluble materials and transferred to a 384-well plate for high throughput analysis using LCMS.

Frozen media samples were thawed on ice and 30µl transferred to a 96-well microplate (Eppendorf) containing 30µL of 100mM ammonium formate, pH 3.0. The microplates were heat sealed, vortexed for 5min at 750 rpm, and centrifuged at 2000 x g for 10 minutes at room temperature. For Hydrophilic Interaction Liquid Chromatography (HILIC) analysis, 25µL of sample was transferred to a new 96 well microplate containing 75µL acetonitrile, whereas samples for C18 analysis were transferred to a new 96-well microplate containing 75µL water (GenPure ultrapure water system, Thermofisher). Each sample solution was transferred to 384-well microplate (Eppendorf) for LCMS analysis.

For each batch, samples were randomized and matrix-matched reference quality controls and batch-specific pooled quality controls were included.

Complex Lipids

Pre-aliquoted EDTA plasma samples (10 µL) were extracted with 30µL of LCMS grade 2-propanol (ThermoFisher) in a 96-well microplate (Eppendorf). Plates were heat sealed, vortexed for 5min at 750 rpm, and centrifuged at 2000 x g for 10 minutes at room temperature. The supernatant (10µL) was carefully transferred to a 96-well plate, leaving behind the precipitated protein. The supernatant was further diluted

with 90µL of 1:3:2 100mM ammonium formate, pH3 (Fischer Scientific): acetonitrile: 2-propanol and transferred to a 384-well microplate (Eppendorf) for lipids analysis using LCMS.

For cell lysates, in a 300 µL, 96 well plate, 10µL supernatant (3:1 isopropanol:ultrapure water) of the extracted cell lysate metabolites was diluted with 90µL of 1:3:2 100mM ammonium formate, pH3: acetonitrile: 2-propanol (Fisher Scientific) and transferred to a 384-well microplate (Eppendorf) for analysis using LCMS.

For each batch, samples were randomized and matrix-matched reference quality controls and batch-specific pooled quality controls were included.

Untargeted Analysis of Primary Metabolites and Biogenic Amines

Untargeted metabolomics analysis was conducted on Waters Acquity™ UPLC system with 2D column regeneration configuration (I-class and H-class) coupled to a Xevo G2-XS quadrupole time-of-flight (qTOF) mass spectrometer. Chromatographic separation was performed using HILIC (Acquity™ UPLC BEH amide, 100 Å, 1.7 µm 2.1× 100mm, , Waters Corporation, Milford, U.S.A) and C18 (Acquity™ UPLC HSS T3, 100 Å, 1.8 µm,, 2.1×100mm, Water Corporation, Milford, U.S.A) columns at 45°C.

Quaternary solvent system mobile phases were (A) 0.1% formic acid in water, (B) 0.1% formic acid in acetonitrile and (D) 100mM ammonium formate, pH 3. Samples were separated using the following gradient profile: for the HILIC separation a starting gradient of 95% B and 5% D was increase linearly to 70% A, 25% B and 5% D over a 5min period at 0.4mL/min flow rate, followed by 1 min isocratic gradient at 100 % A at 0.4mL/min flow rate. For C18 separation, a chromatography gradient of was as follows: starting conditions, 100% A, with linear increase to final conditions of 5% A, 95% B followed by isocratic gradient at 95% B, 5% D for 1 min.

Binary pump was used for column regeneration and equilibration. The solvent system mobile phases were (A1) 100mM ammonium formate, pH 3, (A2) 0.1 % formic in 2-propanol and (B1) 0.1 % formic acid in acetonitrile. The HILIC column was stripped using 90% A2 for 5 min followed by 2 min equilibration using 100% B1 at 0.3 mL/min flowrate. Reverse phase C18 column regeneration was

performed using 95% A1, 5% B1 for 2 min followed by column equilibration using 5% A1, 95% B1 for 5 min.

Untargeted Analysis of Complex Lipids

For the lipidomic assay, untargeted metabolomics analysis was conducted on a Waters Acquity™ UPLC system coupled to a Xevo G2-XS quadrupole time-of-flight (qTOF) mass spectrometer. Chromatographic separation was performed using a C18 (Acquity™ UPLC HSS T3, 100 Å, 1.8 µm, 2.1×100mm, Water Corporation, Milford, U.S.A) column at 55°C. The mobile phases were (A) water, (B) Acetonitrile, (C) 2-propanol and (D) 500 mM ammonium formate, pH 3. A starting elution gradient of 20% A, 30% B, 49% C and 1% D was increased linearly to 10% B, 89% C and 1 % D for 5.5 min, followed by isocratic elution at 10% B, 89%C and 1%D for 1.5 min and column equilibration with initial conditions for 1min.

Mass Spectrometry Data Acquisition

Mass spectrometry data was acquired in sensitivity, positive and negative electrospray ionization mode within 50-1200 Da range for primary metabolites and 100-2000 Da for complex lipids. For the electrospray acquisition, the capillary voltage was set at 1.5 kV (positive), 3.0kV (negative), sample cone voltage 30V, source temperature at 120° C, cone gas flow 50 L/h and desolvation gas flow rate of 800 L/h with scan time of 0.5 sec in continuum mode. Leucine Enkephalin; 556.2771 Da (positive) and 554.2615 Da (negative) for lockspray correction and scans were performed at 0.5 min. The injection volume for each sample was 3µL, unless otherwise specified. The acquisition was carried out will instrument auto gain control to optimize instrument sensitivity over the samples acquisition time.

Pooled quality control samples were analyzed after a defined number of samples to assess replicate precision and allow LOESS correction by injection order. Additional data was captured using the MSe function for pooled quality control samples.

Data Processing

Peak picking and retention time alignment of LC-MS and MSe data were performed using Progenesis QI (Nonlinear, Waters). Data processing and peak annotations were performed using an in-

house automated pipeline. Annotations were determined by matching accurate mass and retention times using customized libraries created from authentic standards and/or by matching experimental tandem mass spectrometry data against the NIST MSMS, LipidBlast or HMDB v3 theoretical fragmentations. To correct for injection order drift, each feature was normalized using data from repeat injections of quality control samples collected every 10 injections throughout the run sequence. Measurement data were smoothed by Locally Weighted Scatterplot Smoothing (LOESS) signal correction (QC-RLSC) as previously described [1]. Only detected features exhibiting a relative standard deviation (RSD) less than 30 in quality control samples were considered for further statistical analysis. To reduce data matrix complexity, annotated features with multiple adducts or acquisition mode repeats were collapsed to one representative unique feature. Features were selected based on replicate precision (RSD<30), intensity and best isotope similarity matching to theoretical isotope distributions. To adjust for inter-site variability, each metabolite was standardized by median-centering the metabolite value for each sample to the median value of the healthy controls.

Enzyme-Linked Immunosorbent Assay

Plasma protein concentrations for CA19-9, LRG1 and TIMP1 were determined as previously described [2]. For all ELISA experiments, each sample was assayed in duplicate and the absorbance or chemiluminescence measured with a SpectraMax M5 microplate reader (Molecular Devices, Sunnyvale, CA). An internal control sample was run in every plate and each value of the samples was divided by the mean value of the internal control in the same plate to correct for interpolate variability.

Statistical Analyses

The AUC that corresponds to the individual performance of all biomarkers is estimated using the area under the empirical estimator of the receiver operating characteristic curve (ROC). The standard error (S.E.) and the corresponding 95% confidence intervals presented for the individual performance of each biomarker were based on the bootstrap procedure in which we re-sampled with replacement separately for

the controls and the diseased 1000 bootstrap samples. We note that for markers LPC(18:0), LPC(20:3) and indole-3-lactate we took into account the inverse directionality, since these markers tend to exhibit higher measurements for the controls compared to the ones that correspond to the cancer related samples. Our model building is based on a logistic regression model using the logit link function. The estimated AUC of the proposed metabolite panel (0.9034) was derived by using the empirical estimator of the linear combination that corresponds to the aforementioned model. The 95% confidence interval we report for the metabolite panel based AUC (0.8180-0.9889) takes into account the fact that the coefficients of the underlying logistic regression model are estimated, and hence exhibit variability, by using the bootstrap with 1000 iterations, for which in every bootstrap iteration the coefficients of the model are re-estimated in order to provide proper inference. Below we present the bootstrap scheme we employ in order to take into account this variability. If we denote with n_x the sample size of the controls and n_y the sample size of the cases the bootstrap based algorithm we used to derive the CIs for the logistic regression based panel during training is the following:

Step 1: Sample with replacement n_x healthy individuals and n_y diseased individuals. Collect for these sampled individuals all scores for each marker that contribute to our marker panel.

Step 2: Based on the samples drawn in Step 1, re-fit the logistic regression model by using the binary disease status as a response and the biomarker scores as covariates.

Step 3: Using the model fitted in Step 2 generate based on its linear part the overall scores from this model for each drawn individual and calculate the AUC.

Step 4: Repeat Steps 1-3 1000 times to collect 1000 values of estimated AUCs, let $\widehat{AUC}_{(b)}$ where b=1,2,...1000.

Step 5: The SE of the original AUC estimate for our data set, let $\widehat{AUC}$, is given by: $SE = \sqrt{\frac{1}{1000-1}\sum_{b=1}^{1000}(\widehat{AUC}_{(b)} - \overline{\mathrm{AUC}})^2}$, where $\overline{\mathrm{AUC}} = \frac{\sum_{b=1}^{1000}(\widehat{AUC}_{(b)})}{1000}$. The corresponding p-value is then calculated based on $Z = \frac{\widehat{AUC}-0.5}{SE(\widehat{AUC})} \sim N(0,1)$.

The protein-metabolite multiplexed panel, i.e. the panel that refers to the combination of the two underlying panels - one for the proteins and one for the metabolites- has been developed using those two panels as two composite markers, considering their respective coefficients fixed (one composite marker for the proteins and one for the metabolites). The protein-metabolite multiplexed panel was developed by combining those two underlying composite markers using a logistic regression model in which we considered the logit link function. The p-values are calculated based on the approximate normality of the 1000 bootstrap based AUC estimates [3]. Wald type CIs are employed using ($\widehat{AUC}$-1.96*SE, $\widehat{AUC}$+1.96*SE) and the corresponding one tailed p-value is provided.

Regarding testing whether an AUC is significant when focusing on an individual marker in the training set, we explored a simpler bootstrap scheme, in which no regression model is involved. More specifically, we consider:

- Step 1: Sample with replacement n_x healthy individuals and n_y diseased individuals.
- Step 2: Using the current bootstrap samples derived from Step 1, calculate the current AUC, let $\widehat{AUC}_{(b)}$.
- Step 3: Repeat Steps 1-3 1000 times to collect 1000 values of estimated AUCs, i.e. $\widehat{AUC}_{(b)}$ where b=1,2,...1000.
- Step 4: The SE of the original AUC estimate of the the studied biomarker, let $\widehat{AUC}$, is given by: $SE = \sqrt{\frac{1}{1000-1}\sum_{b=1}^{1000}(\widehat{AUC}_{(b)} - \overline{\text{AUC}})^2}$, where $\overline{\text{AUC}} = \frac{\sum_{b=1}^{1000}(\widehat{AUC}_{(b)})}{1000}$. The corresponding p-value is then calculated based on $Z = \frac{\widehat{AUC}-0.5}{SE(\widehat{AUC})} \sim N(0,1)$.

Regarding the comparison of two AUCs we need to take into account the underlying correlation. Our bootstrap scheme in this case is of the following form:

- Step 1: Sample with replacement n_x healthy individuals and n_y diseased individuals.

- Step 2: Using the current bootstrap samples derived from Step 1, calculate both AUCs, let $\widehat{AUC}_{(1b)}$ and $\widehat{AUC}_{(2b)}$ for markers 1 and 2 respectively.
- Step 4: Repeat Steps 1-3 1000 times to collect 1000 bootstrap pairs of both estimated AUCs, i.e. $\widehat{AUC}_{(1b)}$ and $\widehat{AUC}_{(2b)}$ where b=1,2,...1000. Let $\mathrm{d}_{(b)} = \widehat{AUC}_{(2b)} - \widehat{AUC}_{(1b)}$ be 1000 bootstrap based values that correspond to the differences of the derived AUCs for each bootstrap iteration.
- Step 5: The p-value for the difference of the initially estimated AUCs of our original samples, i.e. $\widehat{AUC}_{(2)} - \widehat{AUC}_{(1)}$, is then based on:

$$\mathrm{Z} = \frac{\widehat{\mathrm{AUC}}_{(2)} - \widehat{\mathrm{AUC}}_{(1)}}{\sqrt{\mathrm{Var}(\widehat{\mathrm{AUC}}_{(2)}) + \mathrm{Var}\left(\widehat{\mathrm{AUC}}_{(2)}\right) - 2\mathrm{Cov}(\widehat{\mathrm{AUC}}_{(2)}, \widehat{\mathrm{AUC}}_{(1)})}} \sim \mathrm{N}(0,1).$$

where $\mathrm{Var}\left(\widehat{\mathrm{AUC}}_{(\mathrm{k})}\right) = \frac{1}{1000-1}\sum_{\mathrm{b}=1}^{1000}(\widehat{\mathrm{AUC}}_{(\mathrm{kb})} - \overline{\mathrm{AUC}}_{(\mathrm{k})})^2$, where $\overline{\mathrm{AUC}}_{(\mathrm{k})} = \frac{\sum_{\mathrm{b}=1}^{1000}(\widehat{\mathrm{AUC}}_{(\mathrm{kb})})}{1000}$, with k=1, 2. For the derivation of the underlying sample covariance we employ the analogous formula using all 1000 pairs.

For the testing set, all p-values reported for individual marker AUC testing are Wilcoxon based, while for comparing two correlated AUCs the Delong test was employed. We note that for the test set, the coefficients of the trained logistic regression model are considered fixed and known and hence traditional tests can be applied.

References

1. Wang T, Fahrmann JF, Lee H, *et al.* JAK/STAT3-Regulated Fatty Acid beta-Oxidation Is Critical for Breast Cancer Stem Cell Self-Renewal and Chemoresistance. Cell Metab 2018;27(1):136-150.e5.

2. Capello M, Bantis LE, Scelo G, *et al.* Sequential Validation of Blood-Based Protein Biomarker Candidates for Early-Stage Pancreatic Cancer. J Natl Cancer Inst 2017;109(4).

3. Bradley Efron RT. An Introduction to the bootstrap. Chapman and Hall Inc. CRC Press 1993.

Supplementary Table 1. Subject Characteristics in Discovery Cohort #1*

Variables	Pancreatic cancer (EHO)	Healthy controls (EHO)	Healthy controls (MDA)	Chronic pancreatitis (EHO)
Total (n)	20	10	60	10
Gender (n)				
Male	10	4	30	6
Female	10	6	30	4
Mean age (SD)	70.4 (10.0)	60.2 (10.4)	62.0 (4.2)	61.6 (13.3)
Stage (n)				
IB	2	-	-	-
IIA	1	-	-	-
IIB	7	-	-	-
IV	10	-	-	-

***** EHO =Evanston Hospital; MDA = University of Texas, MD Anderson; SD- standard deviation

Supplementary Table 2. Subject Characteristics in Discovery Cohort #2*

Variables	Pancreatic cancer	Low grade pancreatic cyst
Total (n)	9	51
Gender (n)		
Male	3	19
Female	6	32
Mean age (SD)	73.1 (8.1)	62.2 (17.6)
Tobacco smoking		
Never	5	22
Ex-smoker	3	17
Current smoker	1	11
Missing	-	1
Type 2 diabetes		
Yes	4	35
No	5	16
Alcohol drinking		
Never	6	31
Ex-drinker	-	8
Current drinker	3	10
Missing	-	2
Cystic lesion		
IPMN	9	34
MCN	-	11
SCN	-	6
Stage (n)		
IA	1	-
IIA	2	-
IIB	2	-
IV	4	-

*Abbrev. SD- standard deviation; IPMN- intraductal papillary mucinous neoplasm; MCN- mucinous cystic neoplasm; SCN- serous cystic neoplasm

Supplementary Table 3. Subject Characteristics in the Test Sets*

Variables	Test Set #1	
	Pancreatic cancer, No.	Healthy controls, No.
Total	39	82
Gender		
Male	21	43
Female	18	39
Mean age (SD)	62.0 (11.0)	62.8 (10.0)
Tobacco smoking		
Never	16	41
Ex-smoker	12	24
Current smoker	11	17
Alcohol drinking		
Never	23	41
Ex-drinker	9	8
Current drinker	7	32
Missing	-	1
Stage		
IA	6	-
IB	10	-
Resectable (No TNM data)	23	-

*SD- standard deviation; TNM- Tumor (T), Lymph Node (N), Metastasis (M)

Supplementary Table 4. Description of pancreatic cancer cell lines.

Cell line	Type	Origin	Mutational Status				EMT	Gender
			KRAS	p53	CDKN2A	DPC4		
BXPC3	Adenocarcinoma	primary	WT	Mut	HD	HD	Epithelial	Female
SW1990	Adenocarcinoma	spleen met	Mut	WT	HD	ND	Intermediate	Male
SU.86.86	Ductal Carcinoma	liver met	Mut	Mut	HD	WT	Epithelial	Female
PANC-1	Epitheloid Carcinoma	primary	Mut	Mut	HD	WT	Mesenchymal	Male
Hs766T	Carcinoma	lymph node met	Mut	Rearr	Splicing	HD	Intermediate	Male
CFPAC-1	Ductal Adenocarcinoma	liver met	Mut	Mut	Meth	HD	Intermediate	Male
MiaPaCa2	Carcinoma	primary	Mut	Mut	HD	WT	Mesenchymal	Male
AsPC-1	Adenocarcinoma	ascites	Mut	Mut	HD	WT/Mut	Mesenchymal	Female
Panc03.27	Adenocarcinoma	primary	Mut	Mut	HD	HD	Intermediate	Female
HPAF-II	Adenocarcinoma	ascites	Mut	Mut	HD	WT	Epithelial	Male
Capan-2	Adenocarcinoma	primary	Mut	WT/Mut	Mut	WT	Epithelial	Male

*Abbrev: WT- wild type; Mut- mutant; HD- homozygous deletion

Supplementary Table 5. Performance of candidate metabolite biomarkers stratified as early (Stage I-II) and late-stage (Stage IV) in discovery cohorts

Metabolite	Cohort #1: 20 PDAC vs 80 Controls (10 CP + 70 Healthy)		Cohort #2: 9 PDAC vs 51 BPC	
	AUC	P*	AUC	P*
Diacetylspermine	0.8169	<.001	0.7843	.007
Indole-derivative	0.265	.001	0.2767	.03
Lysophosphatidylcholine(18:0)†	0.3431	.03	0.2462	.02
Lysophosphatidylcholine(20:3)	0.305	.007	0.1765	.002
N8-ACETYLSPERMIDINE	0.7675	<.001	0.7124	.04
1-linoleoylglycerol	0.675	.02	0.4815	.87
1-METHYLADENOSINE	0.7631	<.001	0.6797	.09
1-METHYLNICOTINAMIDE	0.355	.05	0.366	.21
2-Arachidonylglycerol	0.6556	.03	0.4118	.41
Acetylcarnitine	0.6613	.03	0.5904	.4
Acylcarnitine(C10:0)	0.6438	.05	0.6296	.22
Acylcarnitine(C14:0)	0.7225	.002	0.5142	.9
Acylcarnitine(C16:0)	0.7475	<.001	0.5359	.74
Acylcarnitine(C18:1)	0.6544	.03	0.5381	.72
Acylcarnitine(C18:2n-6)	0.72	.003	0.5033	.98
Acylcarnitine(C8:0)	0.7113	.004	0.5556	.6
ADENOSINE 5'-MONOPHOSPHATE;AMP	0.2856	.003	0.6187	.26
Alloxan	0.6931	.008	0.4488	.63
ALPHA-D-GLUCOSE	0.8013	<.001	0.5882	.41
Aspartyl-Threonine	0.3413	.03	0.3813	.26
BILIVERDIN	0.74	.001	0.4815	.87
Ceramide(18:0_16:0)	0.7981	<.001	0.3813	.26
Ceramide(34:1)	0.7838	<.001	0.573	.49
Ceramide(42:1)iso	0.65	.04	0.366	.21
Cholesterol Ester(20:5)	0.2819	.003	0.5599	.58
CITRATE	0.6588	.03	0.4771	.84
CORTISOL	0.7912	<.001	0.6776	.09
CORTISONE	0.3194	.01	0.5577	.59
Diacylglycerol(35:1)	0.2419	<.001	0.1394	<.001
GLUTATHIONE	0.225	<.001	0.3072	.07
Hydroxybutyrylcarnitine	0.6988	.006	0.5556	.6
INDOLE-3-ETHANOL	0.0456	<.001	0.3595	.19
LactosylCeramide(30:1);Phosphatidylcholine(38:6)	0.7	.006	0.4728	.8
LactosylCeramide(32:0)	0.3338	.02	0.3682	.21
L-ARGININE	0.7113	.004	0.3464	.15
L-CYSTINE	0.7963	<.001	0.5033	.98
L-PHENYLALANINE	0.7469	<.001	0.3486	.15
L-TRYPTOPHAN	0.7513	<.001	0.2222	.009
L-VALINE	0.7012	.006	0.549	.65
Lysophosphatidylcholine(14:0)	0.3206	.01	0.3747	.24
Lysophosphatidylcholine(17:0)	0.2938	.005	0.3333	.12
Lysophosphatidylcholine(18:3)	0.3013	.006	0.2985	.06
Lysophosphatidylcholine(20:5)	0.3025	.007	0.4292	.51
Lysophosphatidylcholine(24:0)	0.1681	<.001	0.3224	.09
Lysophosphatidylcholine(P-16:0)	0.2994	.006	0.3333	.12
Lysophosphatidylethanolamine(22:6)	0.7025	.005	0.4662	.76
MALTOSE;MELIBIOSE;SUCROSE	0.8644	<.001	0.6536	.15
MALTOSE;MELIBIOSE;SUCROSE	0.8456	<.001	0.512	.92
NG, NG-dimethyl-L-arginine	0.7738	<.001	0.6035	.33
O-decanoylcarnitine	0.7025	.005	0.5577	.59
O-octanoyl-R-carnitine	0.6944	.008	0.4597	.71
Phosphatidylcholine(32:0)	0.7594	<.001	0.5338	.76
Phosphatidylcholine(33:5)	0.84	<.001	0.3943	.32
Phosphatidylcholine(34:2)	0.7038	.005	0.4423	.59
Phosphatidylcholine(38:4)	0.29	.004	0.1634	.001
Phosphatidylcholine(38:5)	0.305	.007	0.3224	.09

Phosphatidylcholine(40:5)	0.2888	.004	0.2985	.06
Phosphatidylcholine(40:8)	0.2419	<.001	0.4118	.41
Phosphatidylcholine(o-42:5) or Phosphatidylcholine(p-42:4) ‡	0.7213	.002	0.6144	.28
Phosphatidylethanolamine(36:4)	0.7475	<.001	0.5185	.87
Phosphatidylethanolamine(37:4)	0.245	<.001	0.4488	.63
Phosphatidylethanolamine(38:4)	0.7069	.004	0.4575	.69
Phosphatidylethanolamine(o-36:5) or Phosphatidylethanolamine(p-36:4)	0.1969	<.001	0.4641	.74
Phosphatidylethanolamine(o-38:5) or Phosphatidylethanolamine(p-38:4)	0.1694	<.001	0.4052	.37
Phosphatidylethanolamine(o-40:7) or Phosphatidylethanolamine(p-40:6)	0.3319	.02	0.2179	.008
piperine	0.2769	.002	0.4684	.77
Plas_Phosphatidylcholine(o-34:3) or Plas_Phosphatidylcholine(p-34:2)	0.3344	.02	0.5861	.42
Plas_Phosphatidylethanolamine(p-18:0_20:4)	0.2644	.001	0.5948	.37
Sphingomyelin(38:1)	0.265	.001	0.2222	.009
Sphingomyelin(39:2)	0.2175	<.001	0.3791	.25
Sphingomyelin(40:2)	0.7862	<.001	0.3508	.16
Sphingomyelin(42:1)	0.1975	<.001	0.2092	.006
Triacylglycerol(16:0_18:1_18:2)	0.6588	.03	0.4292	.51
Triacylglycerol(54:3)	0.7	.006	0.6253	.24
Triacylglycerol(56:7)	0.6788	.01	0.5512	.63
Triacylglycerol(57:3)	0.6681	.02	0.4597	.71
Triacylglycerol(58:9)	0.7144	.003	0.6209	.25
Triacylglycerol(60:5)	0.7163	.003	0.427	.49
TYRAMINE	0.7238	.002	0.342	.14

* P values were calculated using a two-sided Wilcoxon rank-sum test. † Values in parentheses (e.g. (18:0)) represent the number of carbon atoms (i.e. 18) and number of double bonds (0) in the fatty acyl chain of the respective lipid. ‡ Designation between p- and o- refers to whether the respective ether lipid is a plasmanyl- or plasmenyl-phospholipid, respectively.

Supplementary Table 6. Performance of candidate metabolite biomarkers stratified as early (Stage I-II) and late-stage (Stage IV) in discovery cohorts

Metabolite	Cohort #1: 10 Early Stage PDAC vs 80 Controls (10 CP + 70 Healthy)		Cohort #1: 10 Late Stage PDAC vs 80 Controls (10 CP + 70 Healthy)		Cohort #2: 5 Early Stage PDAC vs 51 BPC		Cohort #2: 4 Late Stage PDAC vs 51 BPC	
	AUC	P*	AUC	P*	AUC	P*	AUC	P*
Diacetylspermine	0.855	<.001	0.7788	.004	0.6902	.17	0.902	.008
Indole-derivative	0.2587	.01	0.2713	.02	0.1843	.02	0.3922	.49
Lysophosphatidylcholine(18:0)†	0.2988	.04	0.3875	.25	0.1725	.02	0.3382	.29
Lysophosphatidylcholine(20:3)	0.325	.07	0.2850	.03	0.0863	.003	0.2892	.17
N8-ACETYLSPERMIDINE	0.73	.02	0.8050	.002	0.6745	.21	0.7598	.09
1-linoleoylglycerol	0.5912	.35	0.7588	.008	0.3608	.31	0.6324	.39
1-METHYLADENOSINE	0.7588	.008	0.7675	.006	0.6902	.17	0.6667	.28
1-METHYLNICOTINAMIDE	0.245	.009	0.4650	.72	0.3333	.23	0.4069	.55
2-Arachidonylglycerol	0.6925	.05	0.6188	.23	0.1725	.02	0.7108	.17
Acetylcarnitine	0.7238	.02	0.5987	.31	0.4863	.93	0.7206	.15
Acylcarnitine(C10:0)	0.6013	.3	0.6863	.06	0.6118	.42	0.652	.32
Acylcarnitine(C14:0)	0.8187	.001	0.6262	.2	0.4078	.51	0.6471	.34
Acylcarnitine(C16:0)	0.7812	.004	0.7138	.03	0.4	.47	0.7059	.18
Acylcarnitine(C18:1)	0.7	.04	0.6088	.27	0.3804	.39	0.7353	.12
Acylcarnitine(C18:2n-6)	0.7438	.01	0.6963	.04	0.4039	.49	0.6275	.41
Acylcarnitine(C8:0)	0.7313	.02	0.6913	.05	0.5451	.75	0.5686	.66
ADENOSINE 5'-MONOPHOSPHATE;AMP	0.17	<.001	0.4013	.31	0.6078	.44	0.6324	.39
Alloxan	0.6338	.17	0.7525	.01	0.4941	.98	0.3922	.49
ALPHA-D-GLUCOSE	0.8	.002	0.8025	.002	0.6118	.42	0.5588	.71
Aspartyl-Threonine	0.2263	.005	0.4563	.66	0.3882	.42	0.3725	.41
BILIVERDIN	0.7912	.003	0.6888	.05	0.3961	.46	0.5882	.57
Ceramide(18:0_16:0)	0.8187	.001	0.7775	.005	0.3804	.39	0.3824	.45
Ceramide(34:1)	0.7938	.003	0.7738	.005	0.5255	.86	0.6324	.39
Ceramide(42:1)iso	0.6713	.08	0.6288	.19	0.251	.07	0.5098	.96
Cholesterol Ester(20:5)	0.1988	.002	0.3650	.17	0.4863	.93	0.652	.32
CITRATE	0.6675	.09	0.6500	.13	0.3843	.4	0.5931	.55
CORTISOL	0.9025	<.001	0.6800	.07	0.7608	.06	0.5735	.64
CORTISONE	0.315	.06	0.3237	.07	0.5608	.67	0.5539	.73
Diacylglycerol(35:1)	0.2288	.005	0.2550	.01	0.1216	.006	0.1618	.03
GLUTATHIONE	0.305	.05	0.1450	<.001	0.3686	.34	0.2304	.08
Hydroxybutyrylcarnitine	0.7525	.01	0.6450	.14	0.4235	.59	0.7206	.15
INDOLE-3-ETHANOL	0.0613	<.001	0.0300	<.001	0.3176	.19	0.4118	.57
LactosylCeramide(30:1);Phosphatidylcholine(38:6)	0.6975	.04	0.7025	.04	0.3098	.17	0.6765	.25
LactosylCeramide(32:0)	0.4125	.37	0.2550	.01	0.3176	.19	0.4314	.66
L-ARGININE	0.6562	.11	0.7663	.006	0.1843	.02	0.549	.76
L-CYSTINE	0.7863	.003	0.8063	.002	0.349	.27	0.6961	.2
L-PHENYLALANINE	0.7325	.02	0.7613	.007	0.3843	.4	0.3039	.2
L-TRYPTOPHAN	0.8475	<.001	0.6550	.11	0.1255	.006	0.3431	.31
L-VALINE	0.6863	.06	0.7163	.03	0.5451	.75	0.5539	.73
Lysophosphatidylcholine(14:0)	0.3237	.07	0.3175	.06	0.2471	.07	0.5343	.83
Lysophosphatidylcholine(17:0)	0.2325	.006	0.3550	.14	0.1961	.03	0.5049	.99
Lysophosphatidylcholine(18:3)	0.28	.02	0.3225	.07	0.2314	.05	0.3824	.45
Lysophosphatidylcholine(20:5)	0.2525	.01	0.3525	.13	0.2706	.1	0.6275	.41

Lysophosphatidylcholine(24:0)	0.135	<.001	0.2013	.002	0.2118	.04	0.4608	.81
Lysophosphatidylcholine(P-16:0)	0.2525	.01	0.3463	.12	0.1569	.01	0.5539	.73
Lysophosphatidylethanolamine(22:6)	0.73	.02	0.6750	.07	0.3059	.16	0.6667	.28
NG, NG-dimethyl-L-arginine	0.81	.002	0.7375	.02	0.4706	.84	0.7696	.08
O-decanoylcarnitine	0.735	.02	0.6700	.08	0.4902	.95	0.6422	.36
O-octanoyl-R-carnitine	0.6975	.04	0.6913	.05	0.451	.73	0.4706	.86
Phosphatidylcholine(32:0)	0.7775	.005	0.7413	.01	0.3882	.42	0.7157	.16
Phosphatidylcholine(33:5)	0.8325	<.001	0.8475	<.001	0.251	.07	0.5735	.64
Phosphatidylcholine(34:2)	0.6975	.04	0.7100	.03	0.349	.27	0.5588	.71
Phosphatidylcholine(38:4)	0.2563	.01	0.3237	.07	0.0941	.003	0.25	.1
Phosphatidylcholine(38:5)	0.2687	.02	0.3413	.1	0.2196	.04	0.451	.76
Phosphatidylcholine(40:5)	0.355	.14	0.2225	.005	0.2039	.03	0.4167	.59
Phosphatidylcholine(40:8)	0.2388	.007	0.2450	.009	0.3333	.23	0.5098	.96
Phosphatidylcholine(o-42:5) or Phosphatidylcholine(p-42:4) ‡	0.7375	.02	0.7050	.04	0.3882	.42	0.8971	.009
Phosphatidylethanolamine(36:4)	0.74	.01	0.7550	.009	0.3725	.36	0.701	.19
Phosphatidylethanolamine(37:4)	0.2613	.01	0.2288	.005	0.4078	.51	0.5	1
Phosphatidylethanolamine(38:4)	0.6675	.09	0.7463	.01	0.3137	.18	0.6373	.37
Phosphatidylethanolamine(o-36:5) or Phosphatidylethanolamine(p-36:4)	0.1563	<.001	0.2375	.007	0.2588	.08	0.7206	.15
Phosphatidylethanolamine(o-38:5) or Phosphatidylethanolamine(p-38:4)	0.1525	<.001	0.1863	.001	0.2078	.03	0.652	.32
Phosphatidylethanolamine(o-40:7) or Phosphatidylethanolamine(p-40:6)	0.2825	.03	0.3813	.23	0.1843	.02	0.2598	.12
piperine	0.2687	.02	0.2850	.03	0.3961	.46	0.5588	.71
Plas_Phosphatidylcholine(o-34:3) or Plas_Phosphatidylcholine(p-34:2)	0.22	.004	0.4487	.6	0.4745	.86	0.7255	.14
Plas_Phosphatidylethanolamine(p-18:0_20:4)	0.25	.01	0.2788	.02	0.5529	.71	0.6471	.34
Sphingomyelin(38:1)	0.23	.006	0.3000	.04	0.1216	.006	0.348	.32
Sphingomyelin(39:2)	0.2413	.008	0.1938	.002	0.251	.07	0.5392	.81
Sphingomyelin(40:2)	0.7775	.005	0.7950	.003	0.2157	.04	0.5196	.91
Sphingomyelin(42:1)	0.175	<.001	0.2200	.004	0.149	.01	0.2843	.16
Triacylglycerol(16:0_18:1_18:2)	0.7188	.03	0.5987	.31	0.5176	.91	0.3186	.24
Triacylglycerol(54:3)	0.7388	.01	0.6613	.1	0.6824	.19	0.5539	.73
Triacylglycerol(56:7)	0.6825	.06	0.6750	.07	0.6431	.3	0.4363	.69
Triacylglycerol(57:3)	0.7138	.03	0.6225	.21	0.5451	.75	0.3529	.34
Triacylglycerol(58:9)	0.6675	.09	0.7613	.007	0.5176	.91	0.75	.1
Triacylglycerol(60:5)	0.7338	.02	0.6988	.04	0.5098	.95	0.3235	.25
TYRAMINE	0.6963	.04	0.7513	.01	0.2627	.08	0.4412	.71

* P values were calculated using a two-sided Wilcoxon rank-sum test. † Values in parentheses (e.g. (18:0)) represent the number of carbon atoms (i.e. 18) and number of double bonds (0) in the fatty acyl chain of the respective lipid. ‡ Designation between p- and o- refers to whether the respective ether lipid is a plasmanyl- or plasmenyl-phospholipid, respectively.

Supplementary Table 7. Youden-based cutoffs and associated sensitivity and specificity for the protein-, metabolite-, and protein-metabolite multiplexed panel in the test set*

Panels	Youden index(J)	95% CI of J	Youden based cutoff	95% CI for the Youden based cutoff	Sens at cutoff	Spec at cutoff
**Protein**	0.6337	0.4547-0.7777	30.5920	29.9679-31.2161	0.8462	0.7875
**Metabolite**	0.6436	0.4661-0.7699	-0.9282	-2.1335 - 0.2770	0.7436	0.9000
**Protein-Metabolite Multiplexed panel**	0.7724	0.6069-0.8636	38.6085	37.6223-39.5494	0.8974	0.8750

*CI= confidence interval; Sens = sensitivity; Spec = specificity.

Supplementary Figures

Supplementary Figure 1. Individual AUCs for detected lysophosphatidylcholines, phosphatidylcholines, sphingomyelins, diacylglycerols and ceramides in the discovery cohorts. AUC = area under the curve.

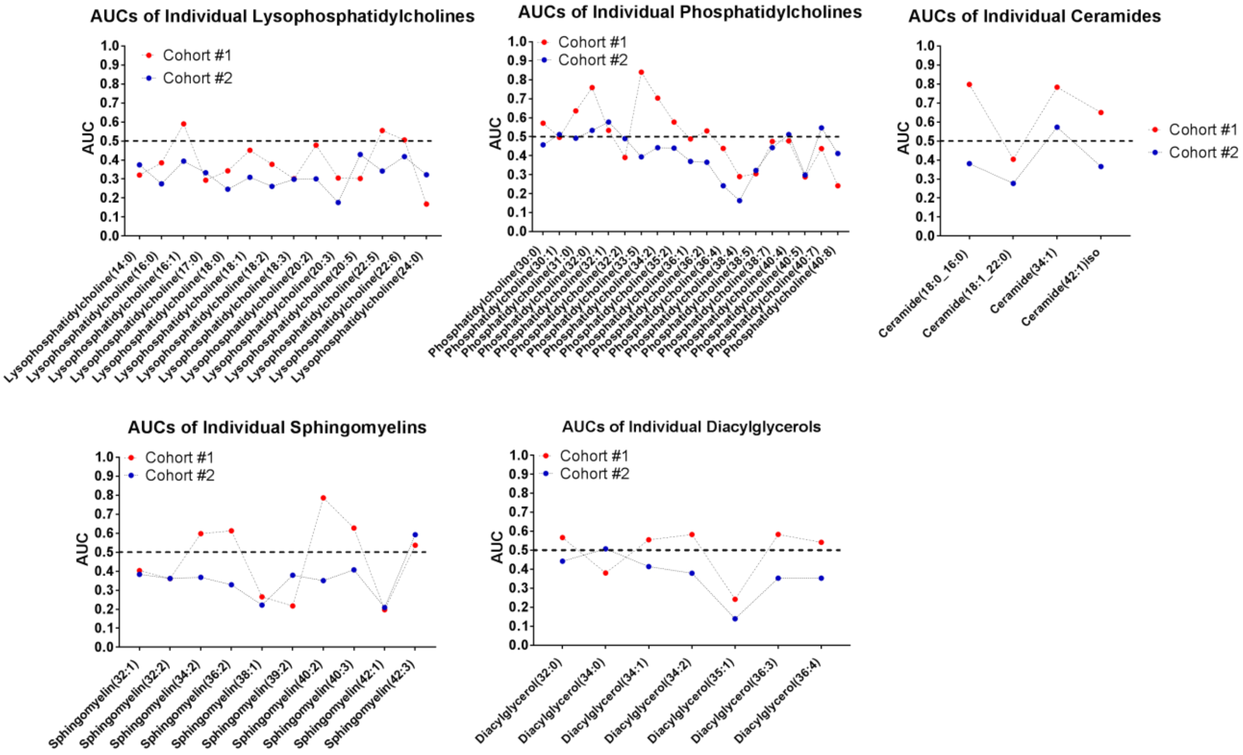

Supplementary Figure 2. Tandem mass spectrometry spectra for indole-derivative.

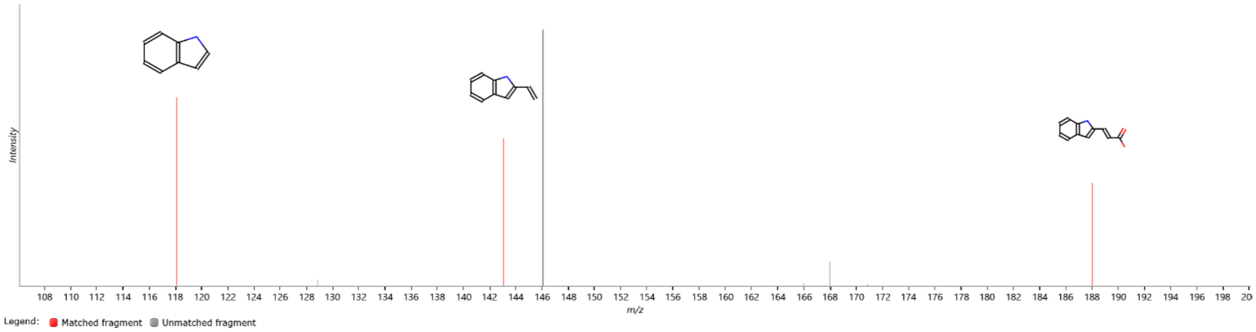

Supplementary Figure 3. Composition of lipid species in conditioned media. Heatmap depicting % change in composition of lipid species in 24, 48 and 72 hour conditioned serum-containing media from PDAC cell lines PANC-1 and SU8686 as compared to media blank. PC = phosphatidylcholine; PE = phosphatidylethanolamine; LPC = lysophosphatidycholine; LPE = lysophosphatidylethanolamine; Plas = Plasmalogen.

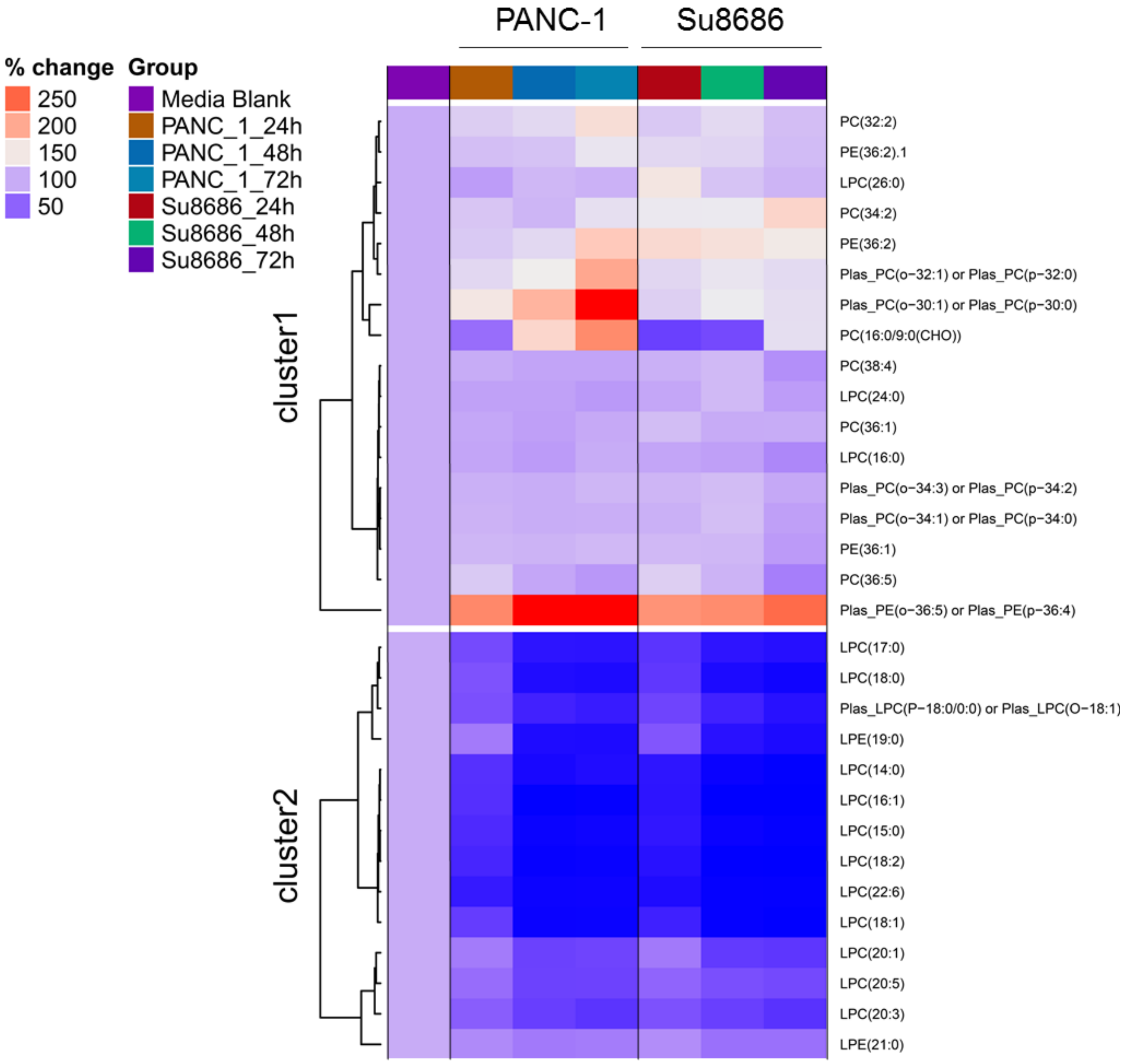

Supplement: Supplementary Data [file djy126_supp.pdf]
